# Improved recombinant expression of soluble cathepsin B and L in *Escherichia coli*

**DOI:** 10.1007/s00253-024-13374-1

**Published:** 2024-12-16

**Authors:** Christina Möller, Niklas Rimkus, Ferdinand F. O. Skala, Maëlle Merouze, Dominique Böttcher, Mark Dörr, Uwe T. Bornscheuer

**Affiliations:** Institute of Biochemistry, Department of Biotechnology and Enzyme Catalysis, University of Greifswald, Greifswald, Germany

**Keywords:** Cathepsin B, Cathepsin L, *E. coli* SHuffle® T7 Express, Recombinant gene expression

## Abstract

**Abstract:**

Cysteine cathepsins such as cathepsin B and L play an important role in numerous diseases like acute pancreatitis or SARS-CoV-2 and therefore have high potential for the development of new therapeutics. To be able to screen for potent and selective inhibitors sufficient amounts of protein are required. Here, we present an easy and efficient protocol for the recombinant expression of soluble and active murine cathepsin B and L. For this, we used the strain *E. coli* SHuffle® T7 Express which is capable of forming disulfide bridges in the cytoplasm. The enzymes were purified by immobilized nickel ion-affinity chromatography. Using different constructs and media, expression levels were significantly improved and expression yields of 80 ± 2 mg L^−1^ for procathepsin B, which is 16-fold better than previously reported expression yields for procathepsin B, and 37 ± 2 mg L^−1^ for procathepsin L, were achieved. After activation with dithiothreitol at slightly acidic pH, in vitro kinetic parameters of both cathepsins were determined using the commonly used synthetic substrates Arg-Arg-AMC or Phe-Arg-AMC. Moreover, to investigate the impact of the short C-terminal propeptide of procathepsin B, it was deleted by site-directed mutagenesis, the shortened target protein was expressed and purified, activated in vitro, and its activity was similar to the variant bearing this C-terminal propeptide.

**Key points:**

*• Recombinant gene expression of cathepsin B and L in E. coli SHuffle® T7 Express*

*• Soluble cathepsin expression with high expression yields*

*• Investigation of the short C-terminal propeptide of cathepsin B*

**Supplementary Information:**

The online version contains supplementary material available at 10.1007/s00253-024-13374-1.

## Introduction

Cathepsins are a family of proteases with a broad range of functions. The cathepsins can be divided into cysteine, serine, and aspartic proteases (Yadati et al. 2020). Papain-like cysteine proteases are ubiquitously expressed and play a role in many physiological and pathological processes (Brix et al. 2008).

Under physiological conditions, cysteine proteases are expressed as preproenzymes, which are inactive precursors, also called “zymogens.” This means that they consist of a signal peptide, a propeptide and a catalytic domain. The signal peptide is responsible for the transport of a protein through membranes into distinct compartments where it is cleaved off by signal peptidases (Von Heijne 1985; Verner and Schatz 1988; Calo and Eichler 2011). The propeptide keeps the protease inactive during the expression to prevent unwanted protein degradation and enable temporal regulation of proteolytic activity (Khan and James 1998; Wiederanders et al. 2003).

For a long time, cysteine proteases have been considered as potential drug targets to be able to treat various diseases (Turk and Gunčar 2003; Bromme 2004). The lysosomal cysteine protease cathepsin B (CTSB) plays a role in the intrapancreatic activation of trypsinogen in the onset of acute pancreatitis (Halangk et al. 2000). Moreover, CTSB has been shown to be up-regulated in a variety of cancers as well as in traumatic brain injury (Hook et al. 2015; Shen and Li 2022). The lysosomal cysteine protease cathepsin L (CTSL) is known to be up-regulated during chronic inflammation and plays a role in the degradation of the extracellular matrix which is important for SARS-CoV-2 to enter host cells. It is also believed to play a role in the processing of the SARS-CoV-2 spike protein (Zhao et al. 2021). Therefore, both cysteine proteases can be considered a possible therapeutic target (Hook et al. 2015; Gomes et al. 2020; Zhao et al. 2021; Shen and Li 2022).

For this reason, there is an increasing interest in cysteine proteases in terms of academic research to unravel their specific functions in various diseases as well as the pharmaceutical interest to be able to find drugs. Consequently, sufficient amounts of protein are required to be able to screen for potent and selective inhibitors and analyse function-structure relationships. Usually, *E. coli* is the first choice for recombinant gene expression since it is a well-established host with short cultivation time, low-cost production, and ease of use (Gopal and Kumar 2013). However, the most commonly used *E. coli* strains such as BL21 (DE3) are not able to form posttranslational modifications like the formation of disulfide bridges in the cytoplasm. It is noteworthy that mature CTSB contains six and CTSL three disulfide bridges, respectively. The recombinant expression of CTSB and CTSL in *E. coli* faced difficulties in soluble expression. Often, the cathepsins were expressed as inclusion bodies which usually require subsequent and time-consuming refolding (Kuhelj et al. 1995; Kramer et al. 2007). Moreover, low expression yields were obtained (Bromme 2004).

Here, we present an easy and efficient protocol for the expression of the murine cysteine proteases cathepsin B and L using *E. coli* SHuffle® T7 Express cells as gene expression system. The cathepsins were expressed as zymogens in a soluble form, purified and activated in vitro. For both cathepsins, we investigated the expression levels using several constructs by varying the location of the His_6_-tag and further optimized the expression of both cathepsins by utilizing different media. Moreover, the very short C-terminal propeptide of cathepsin B was deleted via site-directed mutagenesis in order to investigate its role in comparison to the full-length proprotein.

## Materials and methods

### Molecular cloning

The synthetic genes for the three different variants of murine procathepsin B and the variant of procathepsin L with an N-terminal His_6_-tag were ordered cloned into a pET28a( +) vector (BioCat GmbH, Heidelberg, Germany), where the genes were inserted via seamless cloning using AACTTTAAGAAGGAGATATACC at the 5´site and the XhoI restriction site at the 3´site, respectively. The propeptide sequences were retained, while the signal peptide sequences were excluded in the design of the synthetic genes. The sequences were codon-optimized for expression in *E. coli*. All genes contained an N- or C-terminal His_6_-tag or a His_6_-tag after the sequence for the N-terminal propeptide for purification via affinity chromatography together with an additional cleavage site for the TEV-protease (ENLYFQS) to have the possibility to cleave off the His_6_-tag. The procathepsin L construct CTSL_C-6xHis was generated due to the deletion of the N-terminal His_6_-tag and insertion of a C-terminal His_6_-tag using site-directed mutagenesis. The N-terminal His_6_-tag was deleted using the forward primer 5′-ACCCCGAAATTCGATCAG-3′ and the reverse primer 5′-CATGGTATATCTCCTTCTTAAAG-3′ and the C-terminal His_6_-tag of the pET28a( +) vector was inserted using the forward primer 5′-acttccagagcCTCGAGCACCACCACCA-3′ and the reverse primer 5′-acagattttcATTAACAACCGGATAACTTGCTGC-3′. The amino acid and nucleotide sequences of the variants of procathepsin B and L are listed in the Supplementary Information.

To investigate the function of the C-terminal propeptide of CTSB, the construct CTSB_N-6xHis was used for site-directed mutagenesis where the C-terminal propeptide was deleted. For this variant, the forward primer 5′-GCGCACCGATtagTATTGGGGTC-3′ and the reverse primer 5′-GGAATGCCTGCCACAATTTC-3′ were used.

### Gene expression

For gene expression, the pET28a( +) vector containing the desired gene sequence was introduced into *E. coli* SHuffle® T7 Express cells (New England Biolabs, Frankfurt am Main, Germany) by the heat-shock method. For each gene expression, a single colony of freshly transformed *E. coli* SHuffle® T7 Express cells with the desired gene sequence was picked and used to inoculate 4 mL lysogeny broth (LB) medium supplemented with 50 µg mL^−1^ kanamycin. Subsequently, the cultures were grown overnight at 30 °C at 180 rpm. These starter cultures were used to inoculate the 50 mL main cultures in which the cells were grown. For the main cultures either LB, terrific broth (TB), LB autoinduction medium (LB-AIM), or TB autoinduction medium (TB-AIM) were used. When an optical density (OD_600_) of approximately 0.8–1.0 was reached, the gene expressions of the LB or TB cultures were induced with a final concentration of 0.4 mM isopropyl-β-D-thiogalactopyranoside (IPTG) and were shaken for 26 h at 16 °C at 160 rpm. The LB-AIM and TB-AIM cultures were also shifted to 16 °C for 26 h at 160 rpm when an optical density (OD_600_) of approximately 0.8–1.0 was reached. The main cultures were harvested by centrifugation for 20 min at 4500 × *g* at 4 °C and washed once with sodium phosphate buffer (50 mM, pH 6.0). The cell pellets were stored at − 20 °C until further use. All constructs were expressed three times.

### Cell disruption

The harvested bacteria pellets were resuspended in 4 mL equilibration buffer (50 mM sodium phosphate, 300 mM NaCl, 10 mM imidazole, pH 8.0) for each gram of cell pellet. Afterward, the cells were disrupted using ultrasonication with 50% cycle and 30% power on ice. The sonication procedure consisted of 4 min sonication followed by a 2 min break and another 4 min of sonication. Subsequently, the samples were centrifuged at 10,000 × *g* for 30 min at 4 °C for separation of the cell debris from the supernatant and the lysates were purified.

### Protein purification

Columns of 3 mL Ni-imino diacetate (IDA, Carl Roth, Karlsruhe, Germany) for gravity flow chromatography for the purification of His-tagged proteins were prepared. The columns were washed three times with one column volume of cold MilliQ water followed by five column volume washing steps with equilibration buffer (50 mM sodium phosphate, 300 mM NaCl, 10 mM imidazole, pH 8.0). Afterwards, the clarified lysates containing the desired proteins were transferred onto the Ni-IDA columns, incubated for 30 min on ice, washed 10 times with washing buffer (50 mM sodium phosphate, 300 mM NaCl, 20 mM imidazole, pH 8.0), and eluted in 2 mL fractions with elution buffer (50 mM sodium phosphate, 300 mM NaCl, 250 mM imidazole, pH 8.0). All five elution fractions were pooled.

### Rebuffering of proteins

Prior to protein concentration measurements, the protein samples were rebuffered to remove imidazole in the elution buffer during protein purification. For this, the samples were transferred into centricons with a membrane cutoff of 10 kDa and centrifuged for 20 min at 4500 × *g* at 4 °C and the protein solutions were filled up to 5 mL with CTSB/CTSL measuring buffer (100 mM sodium acetate, 5 mM calcium chloride, pH 5.5). The two steps of centrifugation and filling up with CTSB/CTSL measuring buffer were repeated two more times.

### Protein yields

The protein concentrations were measured at 280 nm via NanoDrop 1000 (Thermo Scientific, Wilmington, DE, USA), and the protein yields were calculated based on the extinction coefficients which were determined using the Expasy tool ProtParam (https://web.expasy.org/protparam/, Table S1).

### Activation of recombinant proteins

The recombinant procathepsins were autocatalytically activated. For this, subsequently after rebuffering, the recombinant procathepsins B and procathepsin L were incubated with 10 mM dithiothreitol (DTT) at 37 °C until fully activated protein could be verified by SDS-PAGE analysis. Precipitated protein was removed by filtration after the activation process.

### Determination of kinetic parameters

The activities of both cathepsins were determined in measuring buffer (100 mM sodium acetate, 5 mM calcium chloride, pH 5.5) using chromogenic substrates. The activity of cathepsin B was measured using the substrate Z-Arg-Arg-AMC (Bachem, Bubendorf, Switzerland), and the activity of cathepsin L was measured using the substrate Z-Phe-Arg-AMC (Bachem). In both cases, the fluorescence was measured at an extinction wavelength of 360 nm and an emission wavelength of 470 nm. *K*_M_ values were determined by measuring initial rates with varying substrate concentrations. For CTSB, the final substrate concentrations of 0, 0.05, 0.1, 0.2, 0.5, 1, 2, and 4 mM of Z-Arg-Arg-AMC were used. For CTSL, the final substrate concentrations of 0, 5, 10, 25, 50, 75, 100, and 150 µM were used. Ninety microliters of the substrate in measuring buffer with the addition of 10 mM DTT were prepared in a reaction plate, and the reaction was started by the addition of 10 µL of a 1 µg mL^−1^ CTSB or CTSL solution. The *K*_M_ values for CTSB were calculated with a nonlinear regression for the fit function for the Michaelis–Menten kinetics and for CTSL with a nonlinear regression using the substrate-inhibition fit in GraphPad Prism 8.4.3 (GraphPad Software, USA) software.

## Results

### Heterologous protein production and purification

For recombinant gene expression of procathepsin B and L in a soluble form and a high expression yield, the *E. coli* SHuffle® T7 Express strain was used. This bacterial strain possesses many advantages for the recombinant expression of eukaryotic proteins as cathepsin B and cathepsin L. It is an engineered *E. coli* strain that is suitable for T7-promoter-based expression and is dedicated to produce correctly folded active proteins containing disulfide bridges within its cytoplasm (Lobstein et al. 2012).

**Fig. 1:**
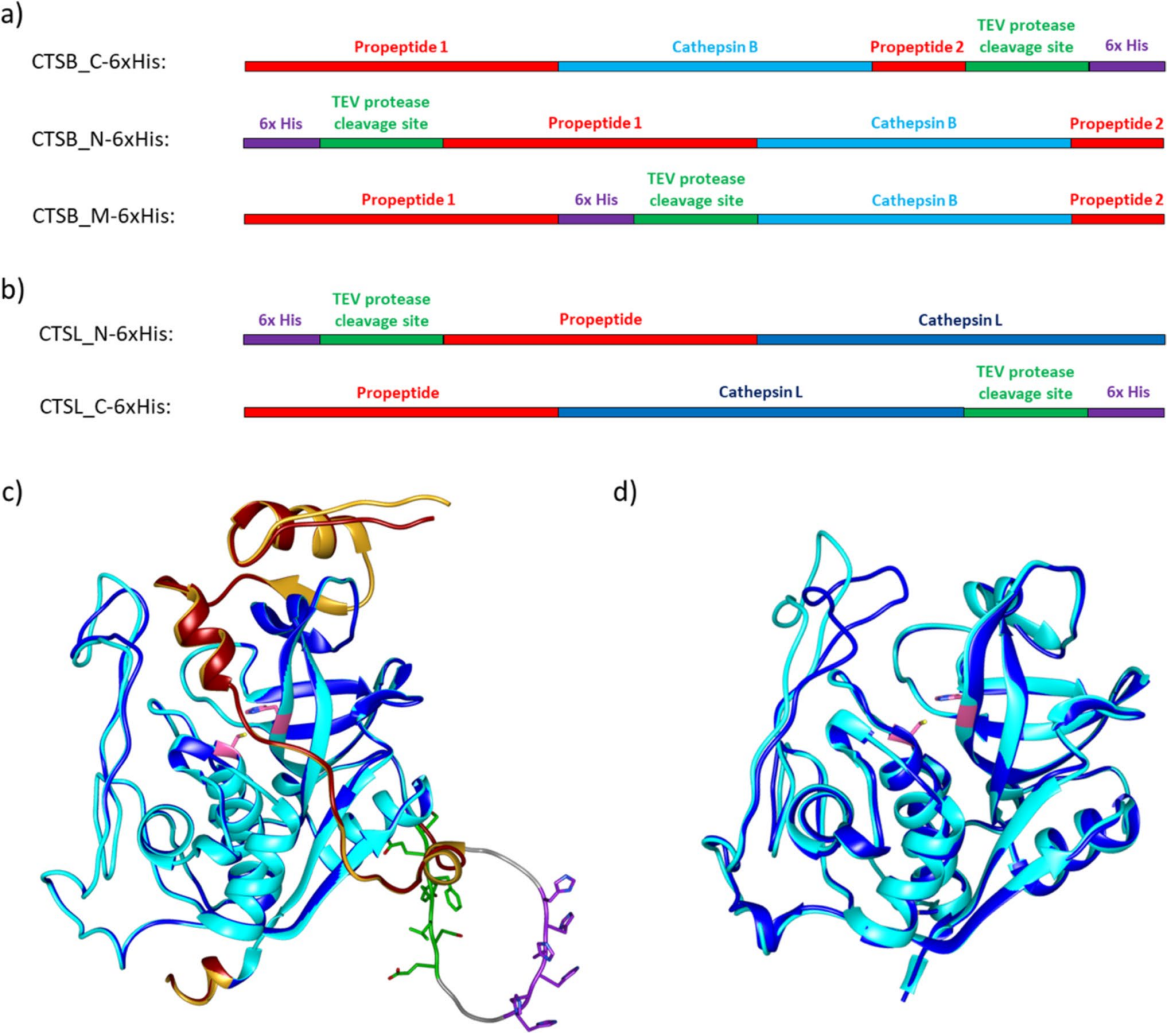
Schematic representations of synthetic genes of **a** procathepsin B (CTSB) and **b** procathepsin L (CTSL). For protein purification, a His_6_-tag with a TEV-protease cleavage site was either added C-terminally (CTSB_C-6xHis, CTSL_C-6xHis), N-terminally (CTSB_N-6xHis, CTSL_N-6xHis), or after the N-terminal propeptide 1 (CTSB_M-6xHis). **c** Overlay of the AlphaFold3 models of CTSB (ptm 0.95) and CTSB_M-6xHis (ptm 0.90) using UCSF chimera. The N-terminal propeptide of CTSB (dark red) and CTSB_M-6xHis (goldenrod) shield the active site cleft with the catalytically active cysteine and histidine residues (pink). The loop where the His_6_-tag (purple) and the TEV-protease cleavage site (green) of CTSB_M-6xHis are located does not seem to interfere with the overall structure of mature CTSB (cyan) compared to CTSB_M-6xHis (dark blue). **d** Overlay of mature human CTSB (PDB-ID: 1CSB) with the murine AlphaFold3 model of CTSB using UCSF chimera. The overall structure of mature human CTSB (dark blue) and murine CTSB (cyan) have a sequence identity of 83% showing overall the same fold. Only minor differences in the loop region of the occluding loop are visible

For gene expression of procathepsin B and L in a soluble form and a high expression yield, several constructs for synthetic genes were designed (Fig. 1a and b). The N-terminal propeptide was maintained since these amino acids shield the active site and therefore inhibit protease activity. Otherwise, the active form of cathepsin B (CTSB) and cathepsin L (CTSL) could result in unwanted protein degradation during recombinant gene expression. Additionally, it was decided to keep the C-terminal propeptide for CTSB since it could be required as well, e.g., for proper protein folding.

In all constructs, a His_6_-tag was added to the protein sequence with an additional cleavage site for the TEV-protease to have the possibility to cleave off the His_6_-tag. The His_6_-tag and TEV-protease cleavage site were either added to the C-terminus (CTSB_C-6xHis, CTSL_C-6xHis), N-terminus (CTSB_N-6xHis, CTSL_C-6xHis) or between the N-terminal propeptide and the mature form of CTSB (CTSB_M-6xHis). The insertion after the N-terminal propeptide would enable the possibility to activate the protein due to the addition of the TEV protease and also to leave the mature protein fused with a His_6_-tag since in both other constructs the His_6_-tag would be cleaved off during autocatalytic activation. Using AlphaFold3 (Abramson et al. 2024) models, it was predicted that the N-terminal propeptide would still correctly occlude the catalytic center and the His_6_-tag would also be accessible for purification (Fig. 1c). The loop where the His_6_-tag and the TEV protease cleavage site were inserted was only enlarged and did not seem to alter the overall protein structure. Moreover, it is shown that mature murine CTSB that has a sequence identity with mature human CTSB of 83% shows an overall similar fold, solely the position of the so called “occluding loop” of CTSB is slightly altered, which is not surprising since it is a flexible loop (Fig. 1d).

**Fig. 2:**
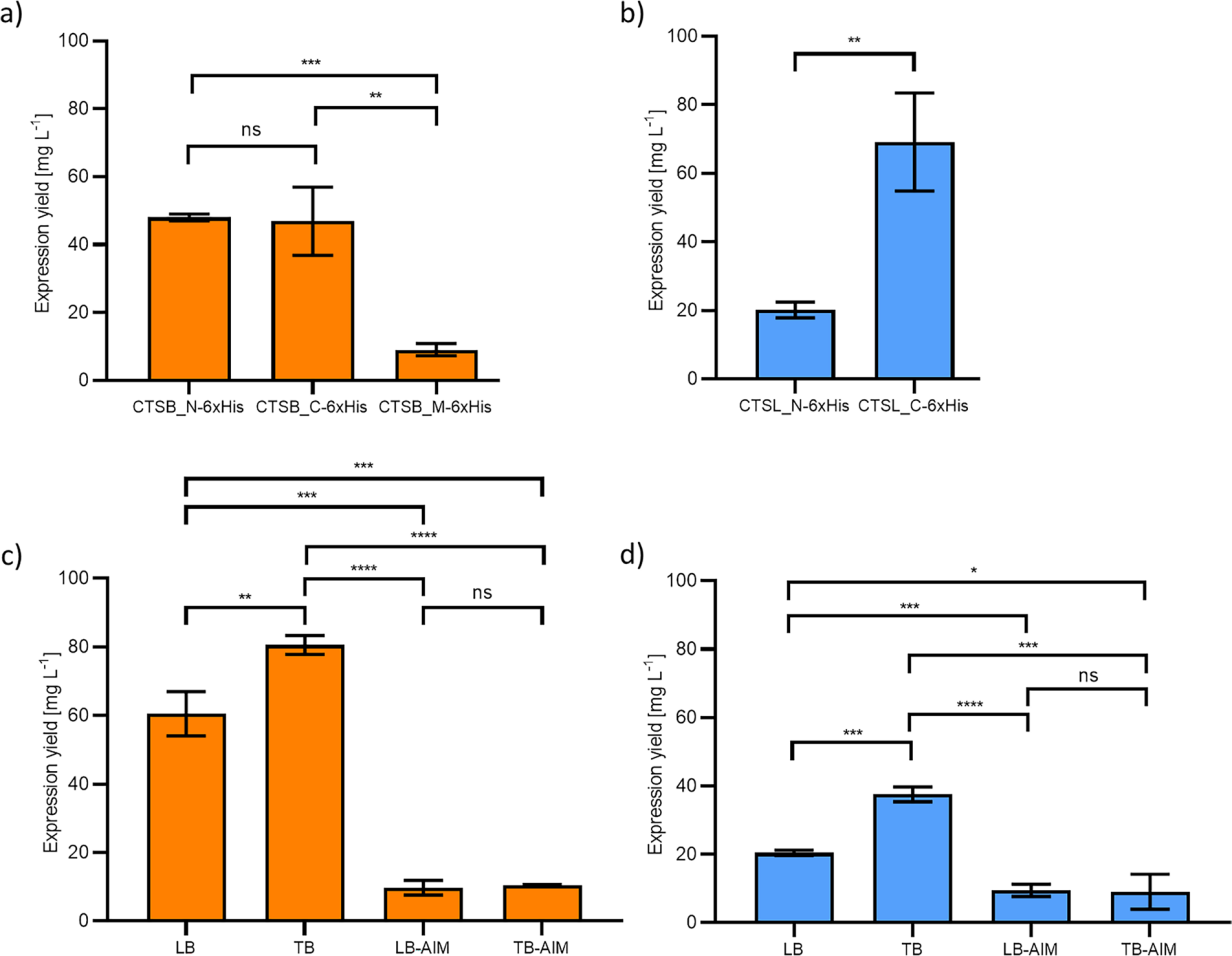
Expression yields of **a** procathepsin B constructs with an N-terminal His_6_-tag (CTSB_N-6xHis), a C-terminal His_6_-tag (CTSB_C-6xHis) and a His_6_-tag after the N-terminal propeptide (CTSB_M-6xHis) expressed in LB-media; **b** procathepsin L (CTSL) constructs with an N-terminal His_6_-tag (CTSL_N-6xHis) and a C-terminal His_6_-tag (CTSL_C-6xHis) expressed in LB medium; **c** procathepsin B containing an N-terminal His_6_-tag (CTSB_N-6xHis) expressed in different media; and **d** procathepsin L containing an N-terminal His_6_-tag (CTSL_N-6xHis) expressed in different media. Media investigated have been lysogeny broth (LB), terrific broth (TB), LB autoinduction medium (LB-AIM), and TB autoinduction medium (TB-AIM). The data represent three independent experiments and the significance was calculated by two-tailed Student *t* test for independent samples

For recombinant protein production, *E. coli* SHuffle® T7 Express cells were used. The highest yields of approx. 50 mg L^−1^ were obtained for the two constructs CTSB_N-6xHis and CTSB_C-6xHis (Fig. 2a). The recombinant expression of CTSB_M-6xHis was also successful with a yield of 9 ± 2 mg L^−1^. All variants were shown to be active (Figure S1). For procathepsin L, the expression of both constructs was also successful (Fig. 2b). Although the expression yield for CTSL_C-6xHis was higher (approx. 70 mg L^−1^) further expression optimization was continued with the CTSL_N-6xHis construct since here the His_6_-tag and cleavage site for the TEV-protease would be completely removed upon activation of the proenzyme. Therefore, mature CTSL_N-6xHis would result in the physiologically relevant enzyme which would not be the case for the construct with the C-terminal His_6_-tag and TEV-protease cleavage site.

To even further increase recombinant protein production, different media were studied (Fig. 2c and d). In both cases, the highest yield was obtained using terrific broth (TB) medium, where yields of 80 ± 2 mg L^−1^ for CTSB_N-6xHis and 37 ± 2 mg L^−1^ for CTSL_N-6xHis were obtained. For expression in lysogeny broth (LB) medium protein amounts of 61 ± 5 mg L^−1^ for CTSB_N-6xHis and 21 ± 1 mg L^−1^ for CTSL_N-6xHis were determined. Low recombinant CTSB and CTSL expression yields were determined using the autoinduction media (AIM) LB-AIM and TB-AIM.

### Biochemical characterization of the proteins

**Fig. 3:**
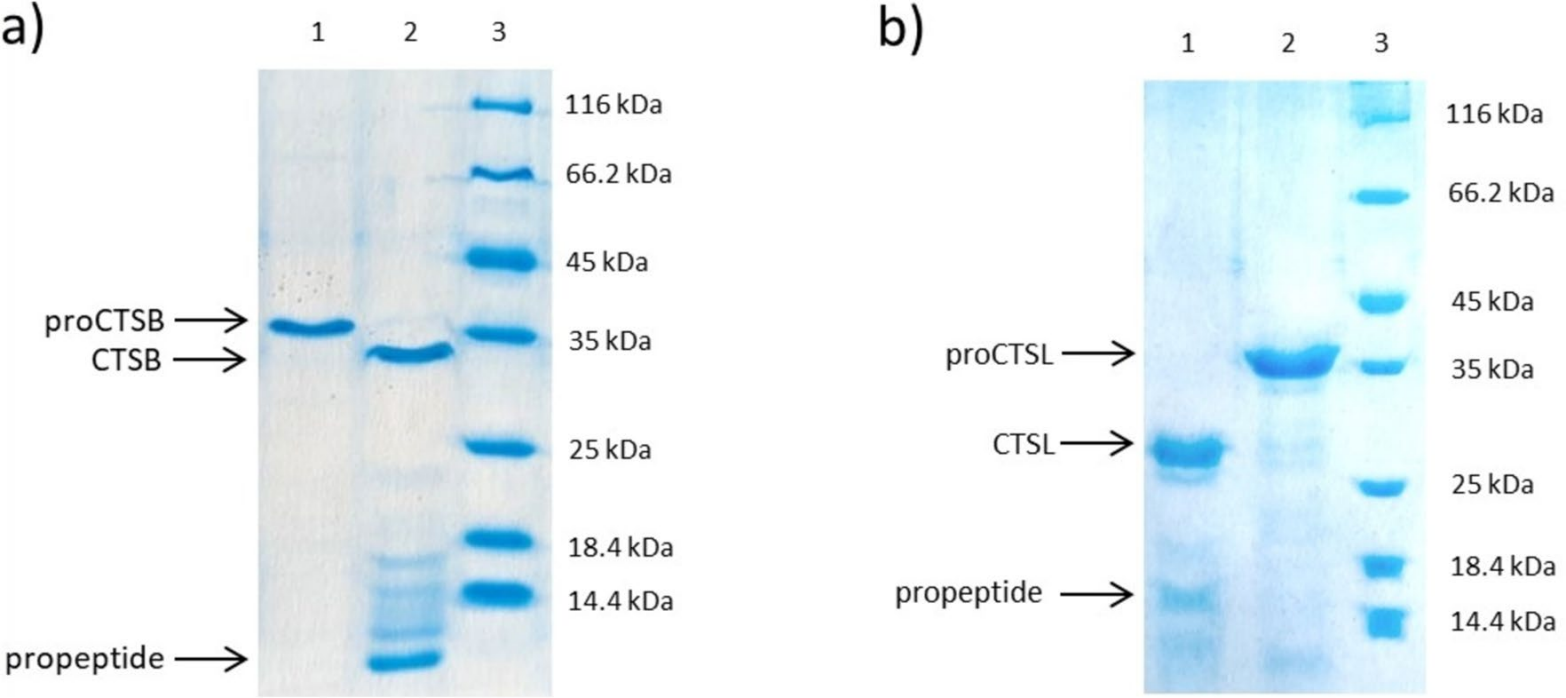
SDS-PAGE analysis of the activation of CTSB and CTSL. **a** Procathepsin B (proCTSB, lane 1), activated CTSB and propeptide (lane 2), and the ladder (lane 3). **b** Activated CTSL and the propeptide (lane 1), procathepsin L (proCTSL, lane 2), and the ladder (lane 3). Pierce™ Unstained Protein MW Marker (ThermoFisher, Germany) was used as a reference

Recombinant procathepsin B and L can be autocatalytically activated by rebuffering in a suitable buffer with a slightly acidic pH value. Both procathepsins were rebuffered in 100 mM sodium acetate buffer supplemented with 5 mM calcium chloride at pH 5.5 and 10 mM dithiothreitol (DTT). The successful activation was verified by SDS-PAGE analysis (Fig. 3). CTSB_N-6xHis has a molecular weight of 37.5 kDa while the mature protein after activation has a molecular weight of 27.6 kDa and the propeptide of 9.1 kDa (Table S1, Fig. 3a). CTSL_N-6xHis has a molecular weight of 38.0 kDa as proenzyme and a molecular weight of 24.0 kDa, when the propeptide with a size of 13.9 kDa is cleaved (Fig. 3b).

To investigate if the C-terminal propeptide is also cleaved in vitro, we separated partially activated CTSB_C-6xHis and mature CTSB_C-6xHis via affinity chromatography (Fig. S2). While the proenzyme was found in the elution fractions, the mature protein did not bind to the column and instead was found in the flowthrough, giving evidence that the C-terminal propeptide is also cleaved off in vitro.

The activity of mature CTSB and CTSL were investigated utilizing the commonly used fluorogenic substrates Z-Arg-Arg-AMC or Z-Phe-Arg-AMC, respectively (Table 1). For CTSB, a K_*M*_ value of 0.37 ± 0.05 mM and a *k*_cat_ value of 77.00 ± 28.90 s^−1^ were obtained. For CTSL, a K_*M*_ value of 0.02 ± 0.00 mM and a *k*_cat_ of 230.00 ± 43.50 s^−1^ were calculated.

**Table 1:** Kinetic parameters for the cathepsin B (CTSB)-catalyzed hydrolysis of Z-Arg-Arg-AMC and the cathepsin L (CTSL)-catalyzed hydrolysis of Z-Phe-Arg-AMC. All experiments were performed with 10 ng CTSB or CTSL in 100 mM sodium acetate buffer with 5 mM calcium chloride at pH 5.5 supplemented with 10 mM DTT

	K_*M*_ (mM)	*k*_cat_ (s^−1^)	*k*_cat_/*K*_*M*_ (mM^−1^ s^−1^)
CTSB	0.37 ± 0.05	77.00 ± 28.90	208.11 ± 33.12
CTSL	0.02 ± 0.00	230.00 ± 43.50	9430.09 ± 337.42

### Determination of the C-terminal propeptide’s function

**Fig. 4:**
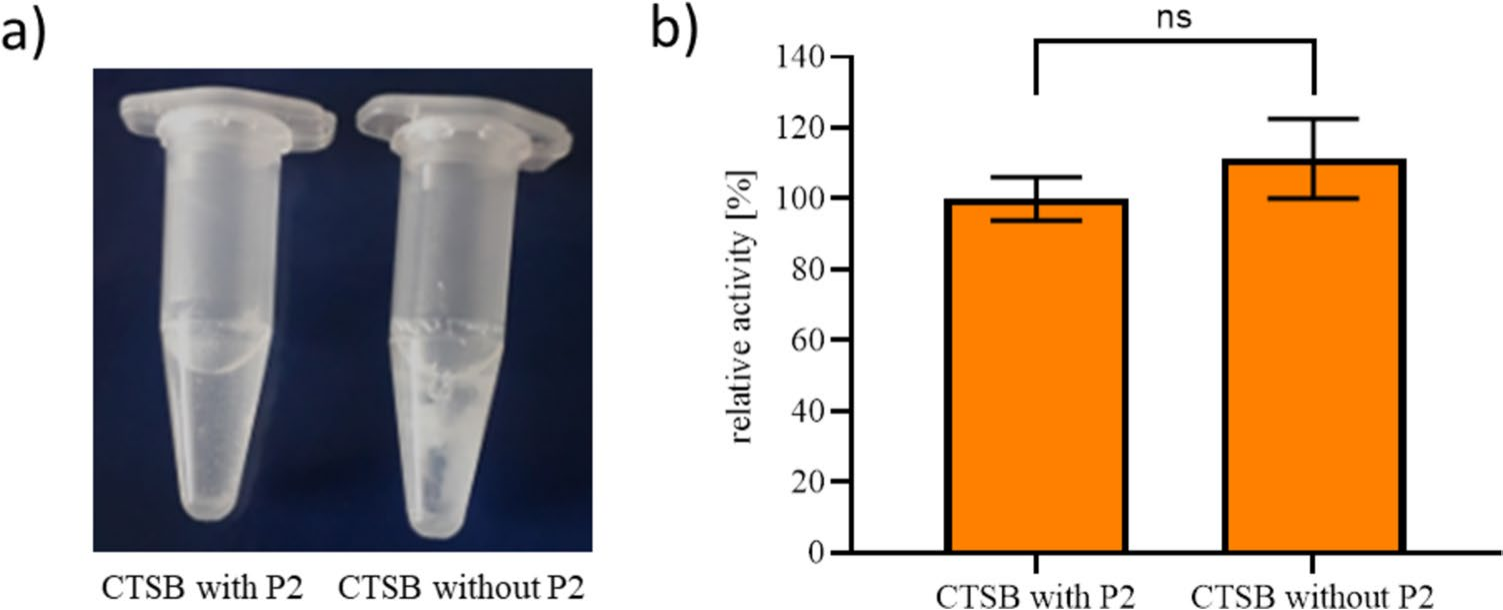
**a** Procathepsin B (CTSB) expressed with the C-terminal propeptide (CTSB with P2) and without (CTSB without P2) after activation at 37 °C. **b** Relative activity of mature CTSB with P2 and CTSB without P2 using Arg-Arg-AMC as a substrate. CTSB with P2 was normalized to 100%, and the relative activity of CTSB without P2 was calculated by dividing the activity measurement by CTSB with P2 and multiply this value with 100 to gain percentage activity. The data represent three independent experiments, and the significance was calculated by two-tailed Student *t* test for independent samples

To investigate the function of the C-terminal propeptide (P2) of CTSB, the C-terminal propeptide of CTSB_N-6xHis was deleted by site-directed mutagenesis and the variants with and without the C-terminal propeptide were expressed and purified. During the activation of both variants, it was observed that parts of the deletion variant precipitated (Fig. 4a). To investigate if CTSB lacking the C-terminal propeptide also influences the activity, activity tests were performed (Fig. 4b). However, no significant difference in the activity of both variants could be observed.

## Discussion

The goal of this study was to achieve an easy and soluble expression of functional cathepsin B and L in the microbial expression host *E. coli* at high expression yields. For the synthesis of recombinant cysteine cathepsins, several protocols were already established, including various expression systems using bacterial, baculovirus, yeast, and mammalian cell culture systems (Hasnain et al. 1992; Kuhelj et al. 1995; Steed et al. 1998; Bromme 2004; Kramer et al. 2007; Zhou et al. 2024). However, these systems are usually quite expensive and time-consuming. Moreover, often challenges as formation of inclusion bodies were faced that are followed by tedious refolding procedures and resulted in low protein yields (Kuhelj et al. 1995; Bromme 2004; Kramer et al. 2007).

Since recombinant protein production in *E. coli* is very easy to do and cost-efficient, we were searching for a suitable *E. coli* expression host that is able to form disulfide bridges, since cathepsin B possesses six and cathepsin L possesses three disulfide bridges (Table S2). Since the most commonly used *E. coli* strains are not capable of the formation of disulfide bridges, we used the strain *E. coli* SHuffle® T7 express. This represents a suitable host for the expression of especially eukaryotic proteins, such as cathepsins since it is able to synthesize correctly folded active enzymes that contain disulfide bridges. It is an engineered *E. coli* strain that can be used for recombinant protein production using a T7-promoter system. This *E. coli* strain is based on a *trxB gor* suppressor strain where its cytoplasmic reductive pathways have been diminished, which enables the formation of disulfide bridges in the cytoplasm. Moreover, a disulfide bond isomerase DsbC was integrated into the chromosome, which corrects the mis-oxidized disulfide bonds (Lobstein et al. 2012).

The recombinant expression of all three cathepsin B variants using *E. coli* SHuffle® T7 Express cells was successful (Fig. 2a). Even though the highest expression yields for procathepsin B were obtained where the His_6_-tag was added either N- or C-terminally, it is worth noting that the His_6_-tag can also be inserted after the N-terminal propeptide. Expression of this construct led to reduced expression yields, however, it was functionally active and could be of interest if a His_6_-tagged version is needed, e.g., if the enzyme has to be removed from a solution after a reaction. Since procathepsin B has a propeptide at both termini, the tag will always be cleaved off if it is added N-terminally or C-terminally. The higher expression yield by using TB medium (Fig. 2c) can easily be explained by the fact that TB medium is an enriched medium that was designed to be able to achieve higher bacterial densities. In 2011, an expression protocol for CTSB expression in soluble form using *E. coli* Rosetta-gami B (DE3) pLysS was already published. However, only low expression yields of 5 mg L^−1^ bacterial culture of the proenzyme were obtained (Novinec et al. 2012).

For the recombinant expression of CTSL, a C-terminally His_6_-tag seems to be the better choice for high expression yields (Fig. 2b). However, since CTSL has no C-terminal propeptide in contrast to CTSB, the His_6_-tag and TEV-protease cleavage site would not be cleaved off upon activation. The His_6_-tag could be cleaved off by using the TEV protease. However, there would still be a six amino acids extension to mature CTSL due to the fact that the TEV-protease cleavage site (ENLYFQS) is cleaved between the amino acids Q and S and therefore six amino acids of the cleavage site would not be cleaved off. To achieve higher yields of physiological CTSL, expression optimization was further carried out with the CTSL_N-6xHis construct where yields of 37 ± 2 mg L^−1^ were obtained (Fig. 2c).

Both procathepsins can be autocatalytically activated by adjusting the pH value to a slightly acidic pH (Fig. 3). The addition of DTT leads to faster full activation for both cathepsins, most likely due to its ability to prevent oxidation of the catalytically active cysteine residue, therefore keeping the proteases in their active state. Moreover, CTSB has a free cysteine residue on the protein surface which could also form an intermolecular disulfide bridge with the free cysteine residue of another CTSB molecule if not prevented.

The activated cathepsin B and L variants showed activity against the common fluorogenic substrates Z-Arg-Arg-AMC and Z-Phe-Arg-AMC, respectively (Table 1). The obtained *K*_*M*_ values for CTSB of 0.37 ± 0.05 mM and 77.00 ± 28.90 s^−1^, respectively, are comparable with the obtained values of the same substrate with recombinantly expressed murine CTSB where values of 0.23 mM and 34 s^−1^ were reported (Caglič et al. 2009). Similar values were also obtained for rat CTSB expressed in yeast where values of 1.27 ± 0.23 mM and 57.9 ± 3.83 s^−1^ were determined (Hasnain et al. 1992). Even though the rat CTSB was expressed in yeast and is therefore glycosylated, this does not seem to alter the activity as it already has been described in the literature (Mach et al. 1992). For CTSL slightly different kinetic parameters compared to the literature were obtained. For CTSL previously expressed in *E. coli* followed by refolding, a *K*_*M*_ value of 1.4 ± 0.1 µM and a *k*_cat_ of 86 s^−1^ were reported. However, these values were determined for human CTSL, which has a sequence similarity of only 77.7% with murine CTSL (Kramer et al. 2007).

Moreover, to investigate the function of the very short C-terminal propeptide of CTSB, this propeptide was deleted using site-directed mutagenesis. During the autocatalytic activation, where the N-terminal and C-terminal propeptides are cleaved off, parts of the variant lacking the C-terminal propeptide precipitated (Fig. 4a). However, the activity of both variants was similar and therefore CTSB lacking the C-terminal propeptide during the activation does not seem to influence the activity and therefore does not lead to alternative protein folding. Since it is known that the C-terminal propeptide does not play a noteworthy role in protein trafficking where it can be considered as a bystander with no recognizable function (Müntener et al. 2003; Bestvater et al. 2005), it can be suggested that this very short propeptide might play a role in protein stability and/or proper protein folding during the expression or activation.

Easy recombinant expression of the cysteine proteases cathepsin B and L is of great interest due to their high potential as drug targets of numerous diseases. The expression protocol reported in this work might also be widely applicable for the recombinant production of other cysteine cathepsins.

## Supplementary Information

Below is the link to the electronic supplementary material.

Supplementary file1 (PDF 745 KB)

## Data Availability

The datasets generated during and/or analyzed during the current study are available from the corresponding author on reasonable request.
