# Supplementary material for: Improved recombinant expression of soluble cathepsin B and L in Escherichia coli
Source: Appl Microbiol Biotechnol. 2024 Dec 16;108:536. doi: 10.1007/s00253-024-13374-1 (PMC11649755; doi:10.1007/s00253-024-13374-1)
Supplement: Supplementary file 1 [file 253_2024_13374_MOESM1_ESM.pdf]

Supplementary Information

Improved Recombinant Expression of Soluble Cathepsin B and L in *Escherichia coli*

Christina Möller[1], Niklas Rimkus[1], Ferdinand F. O. Skala[1], Maëlle Merouze[1], Dominique Böttcher[1], Mark Dörr[1], Uwe T. Bornscheuer[1]*

[1]Institute of Biochemistry, Department of Biotechnology and Enzyme Catalysis, University of Greifswald, Greifswald, Germany

*Corresponding author: E-mail: uwe.bornscheuer@uni-greifswald.de

Amino acid sequences of the synthetic genes of CTSB and CTSL variants

<u>CTSB:</u>

CTSB_N-6xHis (lysosomal protease cathepsin B from mouse with a N-terminal His_6-tag, based on NCBI-code: NP_031824.1):

MHHHHHHGSSGENLYFQSHDKPSFHPLSDDLINYINKQNTTWQAGRNFYNVDISYLKKLCGT
VLGGPKLPGRVAFGEDIDLPETFDAREQWSNCPTIGQIRDQGSCGSCWAFGAVEAISDRTCIHT
NGRVNVEVSAEDLLTCCGIQCGDGCNGGYPSGAWSFWTKKGLVSGGVYNSHVGCLPYTIPP
CEHHVNGSRPPCTGEGDTPRCNKSCEAGYSPSYKEDKHFGYTSYSVSNSVKEIMAEIYKNGPV
EGAFTVFSDFLTYKSGVYKHEAGDMMGGHAIRILGWGVENGVPYWLAANSWNLDWGDNG
FFKILRGENHCGIESEIVAGIPRTDQYWGRF

CTSB_C-6xHis (lysosomal protease cathepsin B from mouse with a C-terminal His_6-tag, based on NCBI-code: NP_031824.1):

MHDKPSFHPLSDDLINYINKQNTTWQAGRNFYNVDISYLKKLCGTVLGGPKLPGRVAFGEDI
DLPETFDAREQWSNCPTIGQIRDQGSCGSCWAFGAVEAISDRTCIHTNGRVNVEVSAEDLLTC
CGIQCGDGCNGGYPSGAWSFWTKKGLVSGGVYNSHVGCLPYTIPPCEHHVNGSRPPCTGEG
DTPRCNKSCEAGYSPSYKEDKHFGYTSYSVSNSVKEIMAEIYKNGPVEGAFTVFSDFLTYKSG
VYKHEAGDMMGGHAIRILGWGVENGVPYWLAANSWNLDWGDNGFFKILRGENHCGIESEI
VAGIPRTDQYWGRFENLYFQSGSSGHHHHHH

CTSB_M-6xHis (lysosomal protease cathepsin B from mouse with His_6-tag after the N-terminal propeptide, based on NCBI-code: NP_031824.1):

MHDKPSFHPLSDDLINYINKQNTTWQAGRNFYNVDISYLKKLCGTVLGGPKLPGRVAFGEDI
DGSSGHHHHHHGSSGENLYFQSLPETFDAREQWSNCPTIGQIRDQGSCGSCWAFGAVEAISDR
TCIHTNGRVNVEVSAEDLLTCCGIQCGDGCNGGYPSGAWSFWTKKGLVSGGVYNSHVGCLP
YTIPPCEHHVNGSRPPCTGEGDTPRCNKSCEAGYSPSYKEDKHFGYTSYSVSNSVKEIMAEIY
KNGPVEGAFTVFSDFLTYKSGVYKHEAGDMMGGHAIRILGWGVENGVPYWLAANSWNLD
WGDNGFFKILRGENHCGIESEIVAGIPRTDQYWGRF

<u>CTSL:</u>

CTSL_N-6xHis (lysosomal protease cathepsin L from mouse with N-terminal His_6-tag, based on NCBI-code: NP_034114.1):

MHHHHHHGMASENLYFQSTPKFDQTFSAEWHQWKSTHRRLYGTNEEEWRRAIWEKNMRMI
QLHNGEYSNGQHGFSMEMNAFGDMTNEEFRQVVNGYRHQKHKKGRLFQEPLMLKIPKSVD
WREKGCVTPVKNQGQCGSCWAFSASGCLEGQMFLKTGKLISLSEQNLVDCSHAQGNQGCN

GGLMDFAFQYIKENGGLDSEESYPYEAKDGSCKYRAEFAVANDTGFVDIPQQEKALMKAVA
TVGPISVAMDASHPSLQFYSSGIYYEPNCSSKNLDHGVLLVGYGYEGTDSNKNKYWLVKNS
WGSEWGMEGYIKIAKDRDNHCGLATAASYPVVN

CTSL_C-6xHis (lysosomal protease cathepsin L from mouse with C-terminal His_6-tag, based on NCBI-code: NP_034114.1):

MTPKFDQTFSAEWHQWKSTHRRLYGTNEEEWRRAIWEKNMRMIQLHNGEYSNGQHGFSME
MNAFGDMTNEEFRQVVNGYRHQKHKKGRLFQEPLMLKIPKSVDWREKGCVTPVKNQGQCG
SCWAFSASGCLEGQMFLKTGKLISLSEQNLVDCSHAQGNQGCNGGLMDFAFQYIKENGGLD
SEESYPYEAKDGSCKYRAEFAVANDTGFVDIPQQEKALMKAVATVGPISVAMDASHPSLQFY
SSGIYYEPNCSSKNLDHGVLLVGYGYEGTDSNKNKYWLVKNSWGSEWGMEGYIKIAKDRD
NHCGLATAASYPVVNENLYFQSLEHHHHHH

Nucleotide sequences of the synthetic genes of CTSB variants and CTSL

<u>CTSB:</u>

CTSB_N-6xHis (lysosomal protease cathepsin B from mouse with a N-terminal His_6-tag, GenBank accession number: PQ417953):

atgcatcatcatcatcaccacggtagcagtggcgaaaatctgtacttccagagccatgataaaccgagcttccatccgctgagtgatgatctgattaatt
atattaacaagcagaacaccacctggcaggcaggccgtaacttctataatgtggatattagttacctgaagaagctgtgtggtaccgttctgggtggtc
cgaaactgccgggtcgtgttgccttcggcgaagatattgatctgccggaaaccttcgatgcccgtgaacagtggagcaattgcccgaccattggcca
gattcgcgatcagggtagctgtggcagctgctgggccttcggcgccgttgaagccattagcgatcgtacctgtattcataccaatggtcgcgtgaatg
ttgaagtgagcgcagaagatctgctgacctgttgtggtattcagtgcggtgatggttgcaatggtggctatccgagcggcgcatggagcttctggacc
aaaaaaggtctggtgagtggtggcgtgtataatagtcatgttggttgtctgccgtataccattccgccgtgtgaacatcatgttaatggcagccgcccg
ccgtgtaccggtgaaggtgataccccgcgttgtaataaaagctgtgaagccggttatagtccgagctataaagaagataaacacttcggctatacca
gttatagtgtgagcaatagcgttaaagaaattatggccgaaatctataagaacggcccggtggaaggcgcattcaccgtgttcagcgacttcctgacc
tataaaagcggcgtgtataaacatgaagcaggcgatatgatgggcggccatgcaattcgtattctgggctggggtgtggaaaatggcgttccgtattg
gctggccgcaaatagctggaatctggattggggcgataatggcttcttcaaaattctgcgtggtgaaaatcattgcggtattgaaagcgaaattgtggc
aggcattccgcgcaccgatcagtattggggtcgcttctaactcgag

CTSB_C-6xHis (lysosomal protease cathepsin B from mouse with a C-terminal His_6-tag, GenBank accession number: PQ417954):

atgcatgataagccgagcttccatccgctgagcgatgatctgattaattatattaacaagcagaacaccacctggcaggcaggccgtaacttctataat
gttgatattagttacctgaagaagctgtgcggcaccgtgctgggtggtccgaaactgccgggtcgcgtggccttcggtgaagatattgatctgccgg
aaaccttcgatgcccgtgaacagtggagtaattgcccgaccattggccagattcgtgatcagggtagttgtggtagctgttgggccttcggcgcagtg
gaagccattagtgatcgtacctgtattcataccaatggtcgtgtgaatgttgaagtgagtgcagaagatctgctgacctgttgcggcattcagtgcggt
gatggttgtaatggtggttatccgagcggcgcatggagcttctggaccaaaaaaggcctggttagcggtggcgtgtataatagtcatgtgggctgcct
gccgtataccattccgccgtgtgaacatcatgttaatggtagtcgcccgccgtgtaccggtgaaggcgataccccgcgctgtaataaaagttgcgaa
gccggctatagtccgagttataaagaagataaacacttcggctataccagctatagcgttagcaatagcgttaaagaaattatggcagaaatctataag
aacggcccggtggaaggtgccttcaccgtgttcagtgacttcctgacctataaaagtggcgtgtataaacatgaagcaggcgatatgatgggtggtc
atgcaattcgcattctgggttggggcgtggaaaatggtgttccgtattggctggccgccaatagttggaatctggattggggcgataatggcttcttca
aaattctgcgcggcgaaaatcattgcggcattgaaagcgaaattgttgcaggtattccgcgtaccgatcagtattggggtcgcttcgaaaatctgtactt
ccagagcggtagcagtggtcatcatcatcatcaccat

CTSB_M-6xHis (lysosomal protease cathepsin B from mouse with His_6-tag after the N-terminal propeptide, GenBank accession number: PQ417955):

Atgcatgataagccgagcttccatccgctgagtgatgatctgattaattatattaacaagcagaacaccacctggcaggcaggtcgtaacttctataat
gttgatattagctacctgaagaagctgtgtggtaccgtgctgggtggcccgaaactgccgggccgtgttgcattcggtgaagatattgatggcagca
gtggccatcatcatcatcaccacggtagcagcggtgaaaatctgtacttccagagcctgccggaaaccttcgatgcacgcgaacagtggagcaatt
gtccgaccattggccagattcgtgatcagggtagctgcggtagttgctgggcattcggcgcagtggaagcaattagtgatcgtacctgcattcatacc

aatggtcgtgttaatgttgaagtgagtgccgaagatctgctgacctgctgtggcattcagtgtggtgatggctgtaatggtggctatccgagtggcgcc
tggagcttctggaccaaaaaaggtctggtgagcggtggcgtgtataatagccatgtgggctgcctgccgtataccattccgccgtgcgaacatcatgt
taatggtagtcgtccgccgtgcaccggcgaaggtgataccccgcgttgtaataaaagttgtgaagccggctatagtccgagttataaagaagataaa
cacttcggctataccagttatagcgttagtaatagtgttaaagagatcatggcagaaatctataaaaacggcccggttgaaggtgcattcaccgtgttca
gcgacttcctgacctataaaagtggcgtgtataaacatgaagccggcgatatgatgggtggccatgccattcgcattctgggttggggcgtggaaaa
tggcgttccgtattggctggccgcaaatagttggaatctggattggggcgataatggcttcttcaaaattctgcgcggtgaaaatcattgcggtattgaa
agcgaaattgttgcaggcattccgcgcaccgatcagtattggggccgcttctaactcgag

CTSL_N-6xHis (lysosomal protease cathepsin L from mouse with N-terminal His_6-tag, GenBank accession number: PQ417956):

Atgcatcatcatcatcaccacggtatggccagcgaaaatctgtacttccagagcaccccgaaattcgatcagaccttcagcgccgaatggcatcagt
ggaaaagtacccatcgccgcctgtatggcaccaatgaagaagaatggcgtcgcgcaatctgggaaaaaaatatgcgcatgattcagctgcataatg
gcgaatatagcaatggtcagcatggcttcagtatggaaatgaatgccttcggtgatatgaccaatgaagagttccgccaggtggttaatggctatcgtc
atcagaaacataaaaaaggtcgcctgttccaggaaccgctgatgctgaaaattccgaaaagtgtggattggcgcgaaaaaggttgcgtgaccccgg
ttaaaaatcagggccagtgcggcagttgctgggccttcagcgccagcggctgcctggaaggccagatgttcctgaaaaccggcaaactgattagtc
tgagtgaacagaatctggttgattgtagtcatgcccagggtaatcagggctgcaatggcggtctgatggacttcgccttccagtatattaaagaaaatg
gtggcctggatagtgaagaaagttatccgtatgaagcaaaagatggcagttgtaaatatcgcgcagaattcgccgtggccaatgataccggcttcgtt
gatattccgcagcaggaaaaagccctgatgaaagccgtggccaccgtgggcccgattagcgtggcaatggatgccagtcatccgagcctgcagtt
ctatagcagtggcatctattatgaaccgaattgcagcagtaaaaatctggatcatggtgtgctgctggttggttatggttatgaaggtaccgatagtaata
aaaataagtactggctggttaagaacagttggggcagcgaatggggtatggaaggctatattaaaattgcaaaagaccgtgataaccattgcggtct
ggccaccgcagcaagttatccggttgttaattaataactcgag

CTSL_C-6xHis (lysosomal protease cathepsin L from mouse with C-terminal His_6-tag, GenBank accession number: PQ417957):

Atgaccccgaaattcgatcagaccttcagcgccgaatggcatcagtggaaaagtacccatcgccgcctgtatggcaccaatgaagaagaatggcg
tcgcgcaatctgggaaaaaaatatgcgcatgattcagctgcataatggcgaatatagcaatggtcagcatggcttcagtatggaaatgaatgccttcg
gtgatatgaccaatgaagagttccgccaggtggttaatggctatcgtcatcagaaacataaaaaaggtcgcctgttccaggaaccgctgatgctgaa
aattccgaaaagtgtggattggcgcgaaaaaggttgcgtgaccccggttaaaaatcagggccagtgcggcagttgctgggccttcagcgccagcg
gctgcctggaaggccagatgttcctgaaaaccggcaaactgattagtctgagtgaacagaatctggttgattgtagtcatgcccagggtaatcaggg
ctgcaatggcggtctgatggacttcgccttccagtatattaaagaaaatggtggcctggatagtgaagaaagttatccgtatgaagcaaaagatggca
gttgtaaatatcgcgcagaattcgccgtggccaatgataccggcttcgttgatattccgcagcaggaaaaagccctgatgaaagccgtggccaccgt
gggcccgattagcgtggcaatggatgccagtcatccgagcctgcagttctatagcagtggcatctattatgaaccgaattgcagcagtaaaaatctgg
atcatggtgtgctgctggttggttatggttatgaaggtaccgatagtaataaaaataagtactggctggttaagaacagttggggcagcgaatggggta
tggaaggctatattaaaattgcaaaagaccgtgataaccattgcggtctggccaccgcagcaagttatccggttgttaatgaaaatctgtacttccaga
gcctcgagcaccaccaccaccaccac

Figures

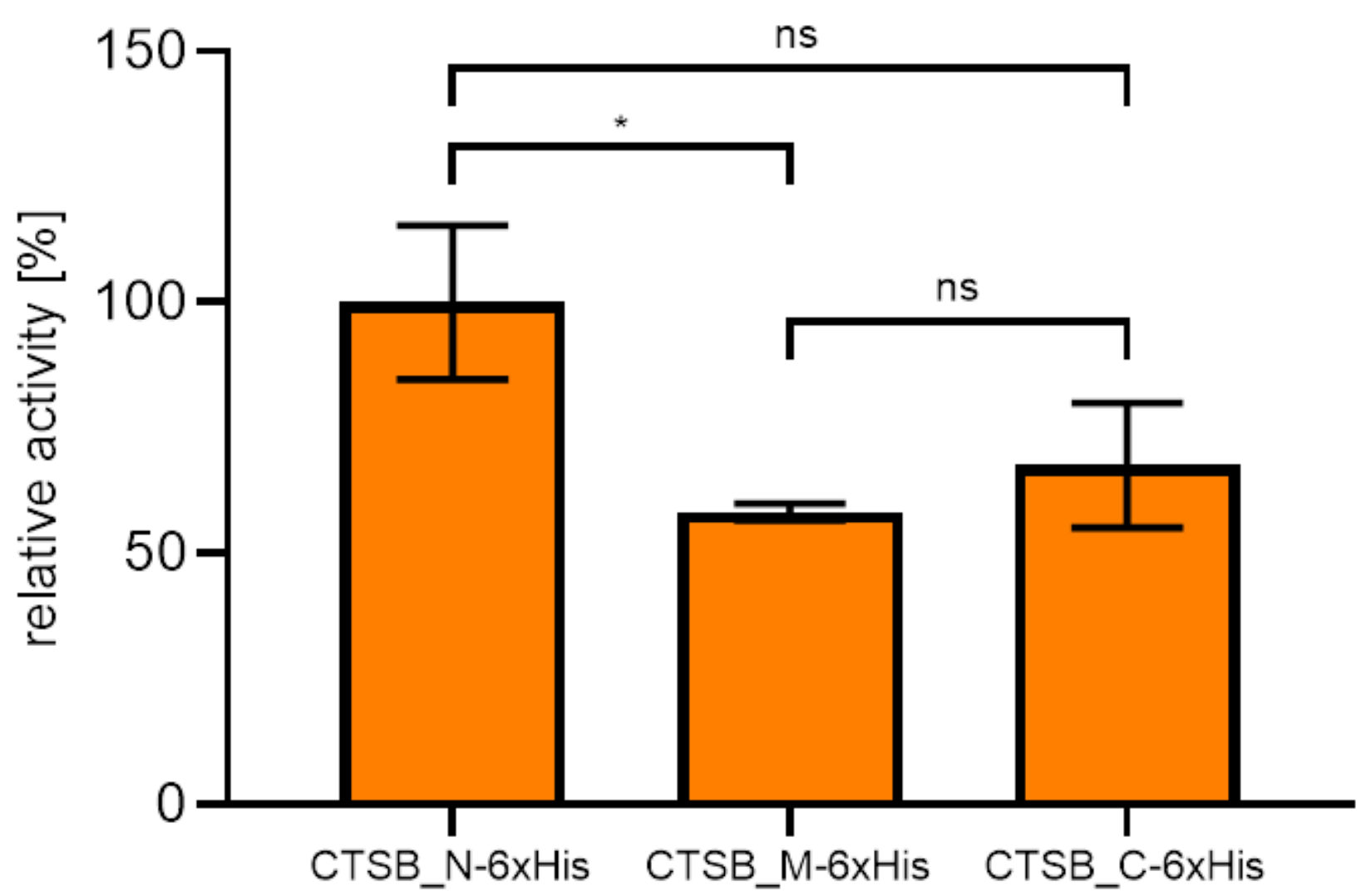

Fig. S1 Relative activities of the mature cathepsin B variants CTSB_N-6xHis, CTSB_M-6xHis and CTSB_C-6xHis using Arg-Arg-AMC as substrate. CTSB_N-6xHis activity was normalized to 100 % and the relative activities of the two other variants were calculated by dividing the data of the variant with the data of CTSB_N-6xHis multiplied with 100 to gain percentage activities. The data represent three independent experiments and the significance was calculated by two-tailed Student t test for independent samples.

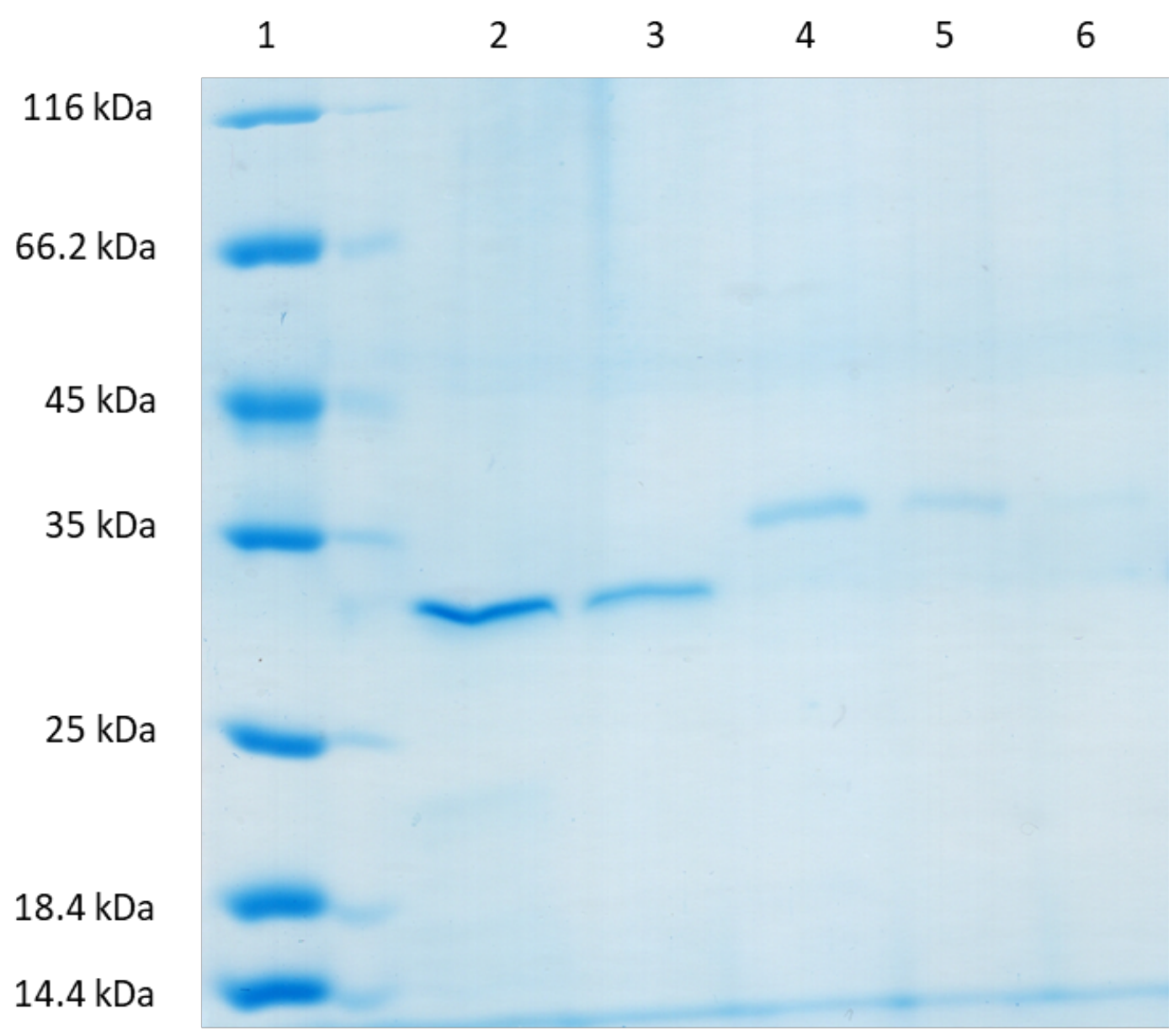

Fig. S2 SDS-PAGE analysis of partly activated CTSB_C-6xHis after separation via affinity chromatography. Shown are the protein marker (lane 1), the flow through (lane 2), wash fraction 2 (lane 3), elution fraction 1 (lane 4), elution fraction 2 (lane 5) and elution fraction 3 (lane 6). PierceTM Unstained Protein MW Marker (ThermoFisher, Germany) was used as a reference.

Tables

Table S1. Overview of the molecular weights (MW) and extinction coefficients (ε) of the cathepsin B (CTSB) and cathepsin L (CTSL) variants calculated via ProtParam. Shown are the MWs of the expressed proCTSB and proCTSL constructs and their corresponding ε, the MWs of the mature cathepsins and their corresponding ε, as well as the MWs of the N-terminal propeptide of CTSB and CTSL and the MWs of the C-terminal propeptide of CTSB.

	MW of proCTSB/ proCTSL [Da]	ε of proCTSB/ proCTSL [M^{-1} cm^{-1}]	MW of mature CTSB/CTSL [Da]	ε of mature variants [M^{-1} cm^{-1}]	MW of the N-terminal propeptide [Da]	MW of the C-terminal propeptide [Da]
**CTSB_N-6xHis**	37500.69	79715	27577.67	61265	9103.10	855.95
**CTSB_C-6xHis**	37500.69	79715	27577.67	61265	7110.05	2849.00
**CTSB_M-6xHis**	37788.95	79715	29858.98	62755	7110.05	855.95
**CTSL_N-6xHis**	38005.50	73840	24080.93	45880	13924.57	–
**CTSL_C-6xHis**	37901.37	73840	26027.99	47370	13924.57	–

Table S2. List of disulfide bridges in murine cathepsin B (CTSB) and murine cathepsin L (CTSL) and their corresponding amino acids.

**proCTSB**	**proCTSL**
Cys76-Cys105	Cys118-Cys161
Cys88-Cys133	Cys152-Cys194
Cys124-Cys190	Cys252-Cys305
Cys125-Cys128	
Cys162-Cys194	
Cys170-Cys181	

Materials and Methods

The activities of cathepsin B variants were determined in 50 mM sodium phosphate buffer pH 6.0 using the chromogenic substrate Z-Arg-Arg-AMC (Bachem, Bubendorf, Switzerland). 10 µl of the cathepsin B variant (0.125 mg mL^{-1}) were prepared in a microtiter plate and the reaction was started by the addition of 90 µL of the AMC substrate solution (2 µM) supplemented with 10 mM DTT. After 5 min incubation, the fluorescence was measured at an extinction wavelength of 360 nm and an emission wavelength of 470 nm.
